# Type 1 Diabetes Mellitus and the First Trimester Placenta: Hyperglycemia-Induced Effects on Trophoblast Proliferation, Cell Cycle Regulators, and Invasion

**DOI:** 10.3390/ijms222010989

**Published:** 2021-10-12

**Authors:** Alejandro Majali-Martinez, Ursula Weiss-Fuchs, Heidi Miedl, Desiree Forstner, Julia Bandres-Meriz, Denise Hoch, Josip Djelmis, Marina Ivanisevic, Ursula Hiden, Martin Gauster, Gernot Desoye

**Affiliations:** 1 Department of Obstetrics and Gynecology, Medical University of Graz, 8036 Graz, Austria; a.majali-martinez@medunigraz.at (A.M.-M.); ursula.weiss-fuchs@ages.at (U.W.-F.); heidi.miedl@meduniwien.ac.at (H.M.); julia.bandres-meriz@medunigraz.at (J.B.-M.); denise.hoch@medunigraz.at (D.H.); ursula.hiden@medunigraz.at (U.H.); 2 Division of Cell Biology, Histology and Embryology, Gottfried Schatz Research Centre for Cell Signaling, Metabolism and Ageing, Medical University of Graz, 8010 Graz, Austria; desiree.forstner@medunigraz.at (D.F.); martin.gauster@medunigraz.at (M.G.); 3 Department of Obstetrics and Gynecology, University Hospital Centre Zagreb, 10000 Zagreb, Croatia; josip.djelmis@pronatal.hr (J.D.); marina.ivanisevic@pronatal.hr (M.I.)

**Keywords:** diabetes, glucose, human trophoblasts, early pregnancy

## Abstract

Type 1 diabetes mellitus (T1DM) is associated with reduced fetal growth in early pregnancy, but a contributing role of the placenta has remained elusive. Thus, we investigated whether T1DM alters placental development in the first trimester. Using a protein array, the level of 60 cell-cycle-related proteins was determined in human first trimester placental tissue (gestational week 5–11) from control (*n* = 11) and T1DM pregnancies (*n* = 12). Primary trophoblasts (gestational week 7–12, *n* = 32) were incubated in the absence (control) or presence of hyperglycemia (25 mM D-glucose) and hyperosmolarity (5.5 mM D-glucose + 19.5 mM D-mannitol). We quantified the number of viable and dead trophoblasts (CASY Counter) and assessed cell cycle distribution (FACS) and trophoblast invasion using a transwell assay. T1DM was associated with a significant (*p* < 0.05) downregulation of Ki67 (−26%), chk1 (−25%), and p73 (−26%). The number of viable trophoblasts was reduced under hyperglycemia (−23%) and hyperosmolarity (−18%), whereas trophoblast invasion was increased only under hyperglycemia (+6%). Trophoblast cell death and cell cycle distribution remained unaffected. Collectively, our data demonstrate that hyperglycemia decreases trophoblast proliferation as a potential contributing factor to the reduced placental growth in T1DM in vivo.

## 1. Introduction

The human placenta is a complex and pleiotropic organ that accommodates both fetal and maternal demands during pregnancy [1]. In the first trimester, placental development relies mainly on the proliferation of villous trophoblasts, a specific cell type of placental villi [2]. Villous trophoblasts can further differentiate along two pathways: (i) they fuse with and expand the syncytiotrophoblast, a multinucleated layer involved in oxygen, nutrient and waste transport, hormone secretion, and protective functions; (ii) they differentiate into extravillous trophoblasts, which invade the decidua and remodel the spiral arteries into low resistance vessels, a process crucial for establishing adequate blood supply to the fetus [3]. Since early in pregnancy the placenta is predominantly exposed to the maternal circulation, these processes are regulated by the maternal metabolic, endocrine, and inflammatory environment [4,5]. Hence, conditions associated with derangements in the maternal milieu, such as obesity and diabetes, affect placental function already at this early period in pregnancy [6,7]. 

Type 1 diabetes mellitus (T1DM) is a disorder characterized by pancreatic dysfunction and β-cell death, ultimately resulting in a dysregulation of the glucose-insulin axis [8]. Due to its rise in incidence, worldwide and among women of reproductive age, the number of pregnancies complicated with T1DM has steadily increased in the last decade [9]. Despite continuous improvement in metabolic control of women during pregnancy, T1DM still entails major adverse consequences for maternal, fetal, and offspring health [10,11]. Although several lines of evidence suggest that T1DM alters fetal development already in the first trimester of pregnancy [12,13], a potential contribution of the placenta has been scarcely investigated.

Some indirect evidence suggested reduced placental growth and/or function in T1DM [14,15]. In this short communication, we, therefore, aimed to determine whether T1DM alters placental proliferation markers and cell cycle regulators in early pregnancy. For that purpose, we have established a unique, well-characterized, but small cohort of first trimester placental tissue from T1DM pregnancies [16,17]. With this tissue available, we could analyze markers of placental growth and cell cycle regulation. Moreover, to further test whether T1DM-associated hyperglycemia contributes to the observed alterations, we also assessed the effect of hyperglycemia on proliferation and invasion in primary human first trimester trophoblasts.

## 2. Results

### 2.1. T1DM Affects Placental Cell Cycle Modulators in the First Trimester of Pregnancy

**Figure 1:**
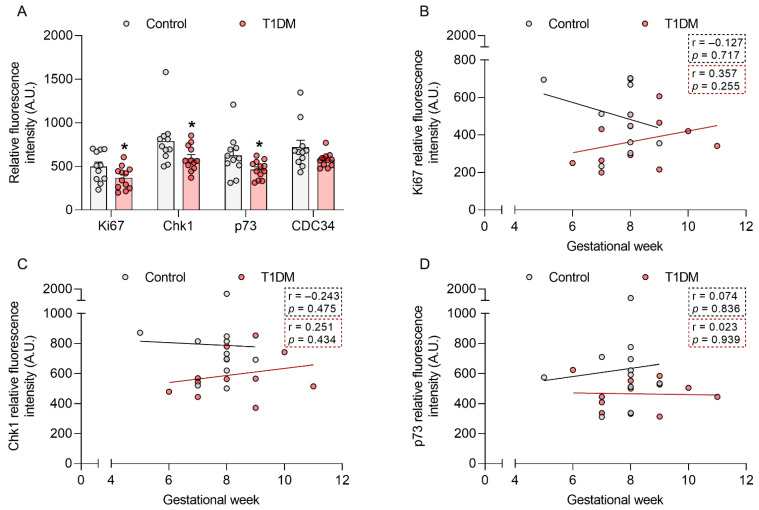
Type-1 diabetes mellitus (T1DM) downregulates Ki67, chk1, and p73 protein levels in early pregnancy. The level of 60 proteins involved in cell cycle regulation and cell proliferation was assessed in human first trimester placental tissue from healthy pregnancies (control, *n* = 11, gestational week (GW) 5–9) and those complicated with T1DM (*n* = 12, GW 6–11) using a protein array (**A**). Associations between GW and Ki67 (**B**), chk1 (**C**), and p73 (**D**) levels were also calculated. Data are presented as mean ± SEM. Statistical analysis used a Mann Whitney U-test and Spearman’s correlation. * *p* < 0.05 vs. control.

To determine whether T1DM compromises cell cycle regulation already in early pregnancy, we assessed the level of 60 proteins related to cell cycle control and cell proliferation in first trimester placental tissue from control pregnancies and those complicated with T1DM (gestational week (GW) 5–11). Protein array results showed a decrease in the protein levels of the proliferation marker Ki67 (−26%; *p* < 0.05) and the cell cycle regulators checkpoint kinase 1 (chk1, −25%; *p* < 0.05), p73 (−26%; *p* < 0.05), and cell division cycle 34 (CDC34, −20%; *p* = 0.09) in T1DM (Figure 1A). 

We have previously shown that gestational age is an important factor to consider when studying early pregnancy [18,19]. Thus, we further stratified the data for those proteins significantly downregulated by T1DM (*p* < 0.05) according to GW (Figure 1B–D). We did not find any significant correlation between Ki67, chk1, and p73 protein levels and GW. However, Ki67 showed a different trend in the control vs. T1DM samples, with a downregulation towards the end of the first trimester in the controls but not in the T1DM cohort.

**Figure 2:**
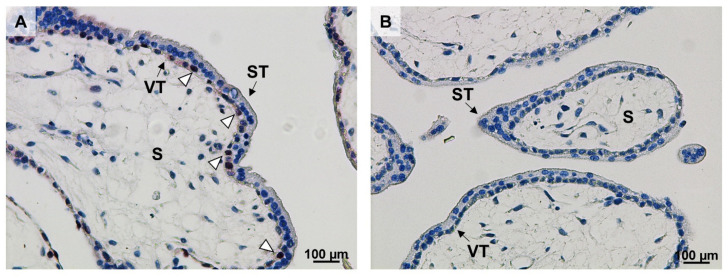
Ki67 localizes to villous cytotrophoblasts (VT) in early pregnancy. Ki67 immunostaining was performed in control first trimester placental tissue (*n* = 1, gestational week 7, (**A**)). Ki67 was predominantly located in the nuclei of VT (open arrowheads). A negative IgG isotype control is shown in (**B**). Scale bar: 100 µm. VT: villous cytotrophoblasts; ST: syncytiotrophoblast; S: stroma.

We subsequently immunolocalized Ki67 in villous tissue of control first trimester placentas. Ki67 was located in the nuclei of villous cytotrophoblast, whereas the syncytiotrophoblast was devoid of immunolabel (Figure 2). Paraffin-embedded first trimester placental tissue was not available from women with T1DM precluding immunolocalization.

### 2.2. Hyperglycemia Decreases Trophoblast Cell Number in Early Pregnancy

**Figure 3:**
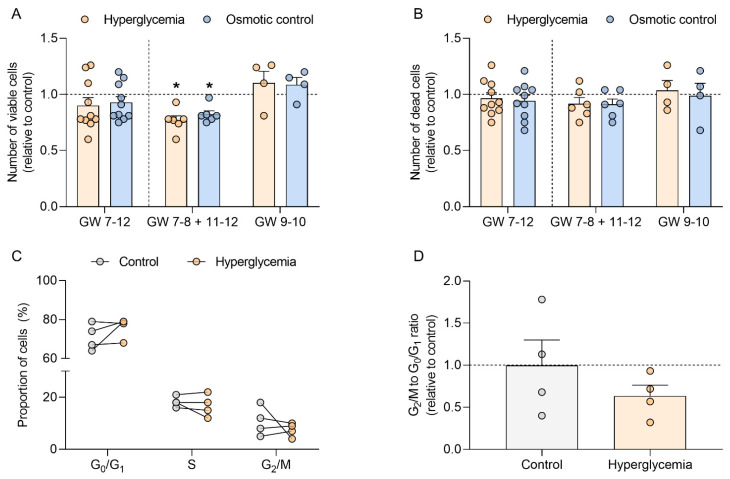
Hyperglycemia decreases viable cell count in human first trimester trophoblasts and does not affect cell death or cell distribution within the cell cycle. Primary human first trimester trophoblasts (gestational week (GW) 7–8, *n* = 8; GW 9–10, *n* = 4; GW 11–12, *n* = 2) were cultured in absence (control) or presence of 25 mM D-glucose (hyperglycemia) and 5.5 mM D-glucose + 19.5 mM D-mannitol (osmotic control) for 48 h. The numbers of viable (**A**) and dead cells (**B**) were counted in duplicates using the CASY TTC Cell Analyzer. The proportion of cells in the different phases of the cell cycle was determined by FACS (**C**). The ratio between the number of cells in G_2_/M and G_0_/G_1_ was also calculated (**D**). Results were calculated relative to the control, arbitrarily set to 1, and presented as mean ± SEM (**A**,**B**,**D**) or expressed as percentage of cells (**C**). Statistical analysis used Wilcoxon signed rank test. * *p* < 0.05 vs. control.

Hyperglycemia is one of the hallmarks of T1DM. Thus, to test whether T1DM-associated hyperglycemia could directly modulate cell cycle progression in early pregnancy, we assessed the number of viable and dead cells and the proportion of cells in each phase of the cell cycle in first trimester trophoblasts (GW 7–12) cultured under normoglycemic (control) and hyperglycemic (25 mM D-glucose) conditions (Figure 3). 

Although the number of viable trophoblasts initially remained unaffected by high glucose concentration (Figure 3A, GW 7–12), data stratification for GW showed a different pattern for samples from early (GW 7–8) and late (GW 11–12) vs. mid first trimester (GW 9–10). Thus, when the results in trophoblast from GW 7–8 and 11–12 were pooled together, hyperglycemia significantly reduced the number of viable cells (−23% vs. control, *p* < 0.05), whereas samples from GW 9–10 showed an opposite trend. Similar results were obtained in the osmotic control (5.5 mM D-glucose + 19.5 mM D-mannitol), which also reduced the number of viable trophoblasts from GW 7–8 and 11–12 (–18% vs. control; *p* < 0.05). However, the number of dead cells was not altered by the treatments and remained unaffected after data stratification according to GW (Figure 3B). 

Hyperglycemia did not alter the proportion of early first trimester trophoblasts (GW 7–8) in the different phases of the cell cycle (Figure 3C). Although we observed a decrease in the G_2_/M to G_0_/G_1_ ratio in trophoblasts cultured under hyperglycemic conditions (–36%; *p* > 0.05), these results did not reach significance (Figure 3D). 

### 2.3. Hyperglycemia Increases Trophoblast Invasion in Early Pregnancy

**Figure 4:**
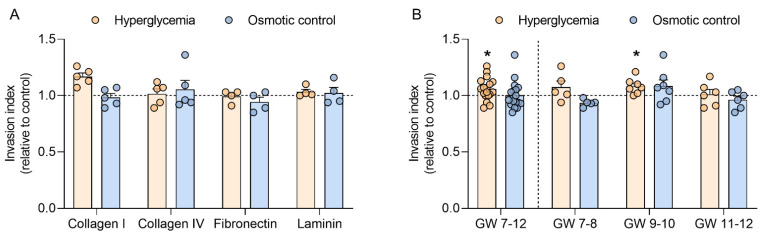
Hyperglycemia increases trophoblast invasion in mid-early pregnancy. Primary human first trimester trophoblasts (gestational week (GW) 7–8, *n* = 5; GW 9–10, *n* = 7; GW 11–12, *n* = 6) were cultured in absence (control) or presence of 25 mM D-glucose (hyperglycemia) and 5.5 mM D-glucose + 19.5 mM D-mannitol (osmotic control) for 48 h. Trophoblast invasion was quantified using a Transwell assay with four different extracellular matrices: collagen I, collagen IV, fibronectin, and laminin (**A**). Invasion data from the different matrices were pooled and also analyzed for different GW periods (**B**). Results were expressed as invasion index and calculated relative to the control, arbitrarily set to 1. Each condition was assayed in quadruplicates. Data are presented as mean ± SEM. Statistical analysis used Wilcoxon signed rank test. * *p* < 0.05 vs. control.

To test whether hyperglycemia can further modulate trophoblast function in the first trimester, trophoblast invasion was determined in four different extracellular matrices, i.e., collagen I, collagen IV, fibronectin, and laminin. When compared to the control, high glucose did not alter trophoblast invasion in the different matrices used. Similar results were obtained in the osmotic control (Figure 4A).

Since trophoblast invasion did not differ between the matrices, data were subsequently pooled and analyzed according to GW (Figure 4B). In this analysis, hyperglycemia significantly upregulated trophoblast invasion in samples spanning GW 7 to 12 (+6%; *p* < 0.05). In a sub-analysis stratifying data in the early (GW 7–8), mid (GW 9–10), and late (GW 11–12) first trimester, the effect of high glucose on trophoblast invasion only reached significance in trophoblasts from GW 9–10 (+8%; *p* < 0.05). No differences were observed in the osmotic control.

## 3. Discussion

Adequate human placental development is required for a successful pregnancy. This is especially critical in the first trimester, when the placenta attains its highest weekly growth rate [20]. Placental development is regulated by the intrauterine environment. Here, we focused on T1DM, a condition characterized by maternal metabolic derangements, and studied its effect on placental cell cycle regulators and cell proliferation in early pregnancy.

We identified three cell-cycle-related proteins that were affected by T1DM: Ki67, chk1, and p73. Ki67 is a classical marker for cell proliferation [21]. In line with earlier results [22], we immunolocalized it to villous cytotrophoblasts, a proliferative trophoblast subpopulation involved in placental villus development and syncytiotrophoblast renewal [23]. Thus, a decrease in Ki67 may reflect reduced trophoblast expansion and, hence, placental growth and function already in early pregnancy. Our data indicate a potential interaction between T1DM and gestational age, which is in concordance with previous reports in diabetic rats, showing that diabetes regulates Ki67 in a time-dependent manner [24]. Interestingly, term placental Ki67 levels are upregulated in gestational diabetes mellitus [25]. Ki67 is a complex molecule with multiple binding domains for interaction partners such as kinesin-like motor protein Hklp2, nucleolar protein NIFK, protein phosphatase 1, and others [21]. However, the presence and location of these proteins in the human first trimester placenta and their role for Ki67 in trophoblast proliferation has remained elusive and will be the topic of future studies.

T1DM was also associated with a downregulation of placental chk1 and p73 protein levels. Chk1 plays a key role in cell cycle control, DNA damage repair, and apoptosis [26], and hyperglycemia impairs chk1 signaling [27]. p73 is a member of the p53 family that regulates the transcription of several genes involved in cell growth and cell cycle progression [28]. Recently, it has been implicated in mouse embryonic development [29]. Thus, chk1 and p73 downregulation is in line with the decrease in Ki67 protein levels and again suggests reduced placental growth in first trimester T1DM. 

Collectively, our data provide molecular evidence supporting earlier speculations of reduced placental growth in early T1DM pregnancies, which were based on indirect evidence [14,15]. Therefore, these results argue for a delay in placental growth in early pregnancy followed by a catch-up growth phase later in T1DM pregnancies. In human, no longitudinal data are available on placental growth in T1DM, but this biphasic placental growth was found in a rat model of T1DM [30]. Since placental growth precedes fetal growth, one can speculate that also fetal growth in T1DM is biphasic, which indeed has been previously observed [31].

Hyperglycemia is considered one of the hallmarks of T1DM [32]. To test whether elevated glucose levels can directly impair progression through the cell cycle of trophoblasts in early pregnancy, we used an established in vitro model of primary first trimester trophoblasts [33] and quantified the number of viable and dead cells. Both high glucose levels and osmotic pressure were associated with a decrease in trophoblast cell number. This is in agreement with our previous findings showing that hyperglycemia and hyperosmolarity decrease cell number and cell proliferation in different first trimester trophoblast models including JAR, JEG-3, and BeWo cells [34]. The results suggest that high-glucose-associated hyperosmolarity impairs trophoblast cell cycle progression in early pregnancy. 

Importantly, the model we used here allowed testing for effects of gestational age. Indeed, GW influenced the results, and the effect of hyperglycemia on viable cell number specifically differed in trophoblasts from GW 9–10 vs. GW 7–8 and 11–12. This is the time window when spiral arteries are remodeled and the intrauterine oxygen tension rises [35]. Thus, these gestational age differences may be attributed to the interplay between oxygen and glucose, since oxygen has been previously shown to modulate cell proliferation in response to hyperglycemia in the first trimester trophoblast cell line ACH-3P [36]. 

The reduction in number of viable cells is not accounted for by an increase in apoptotic cells, since glucose treatment did not alter the number of dead trophoblast cells. Although the proportion of trophoblast cells in each phase of the cell cycle remained unaltered, the G_2_/M to G_1_/G_0_ ratio was slightly downregulated under high glucose treatment, which may reflect changes in the cell cycle resulting in the reduced number of viable cells. More detailed studies with larger samples sizes spanning the whole period of the first trimester of pregnancy are needed. Collectively, these results suggest that hyperglycemia impairs cell cycle completion of trophoblasts in early pregnancy. This in vitro finding may translate into the reduced trophoblast proliferation in T1DM in vivo found above.

Similar to trophoblast proliferation, adequate trophoblast invasion during the first trimester is required for a successful pregnancy. Shallow trophoblast invasion has been linked to pregnancy complications such as preeclampsia and fetal growth restriction [37], whereas abnormal in-depth invasion has been associated with placenta accreta [38]. Here, we showed in vitro an upregulation of trophoblast invasion by hyperglycemia in the first trimester of pregnancy. Although high glucose inhibits cell migration and invasion in HTR-8/SVNeo cells [39,40], we have previously shown that placental matrix metalloproteinase 14, an extracellular matrix-degrading protease crucial for trophoblast invasion, is upregulated by T1DM in early pregnancy [17]. This highlights the importance of using first trimester placental tissue and primary trophoblasts, since results in cell lines do not always reflect the in vivo situation. The effect of high glucose concentrations on trophoblast invasion was more prominent in GW 9–10. It is important to stress that the invasion assay used here allows accounting for potential changes in cell number during the incubation period, since the invasion index as calculated takes into account the total number of both invading and non-invading cells. Thus, the specific effects on invasion described are not accounted for by changes in the cell cycle but reflect a true change in invasion capacity induced by hyperglycemia. Again, further studies need to assess the interplay between oxygen and glucose and its potential effect on fine-tuning trophoblast invasion in early pregnancy in vitro.

To the best of our knowledge, this is the first study assessing placental cell cycle regulation and trophoblast proliferation in T1DM in early pregnancy, a period when placental growth is particularly susceptible to environmental perturbations [20]. The major strength of this study is the use of a unique cohort of human placental tissue from the first trimester of T1DM pregnancies as well as primary first trimester trophoblasts. However, the small T1DM cohort and, in general, the limited availability of first trimester material precluded an in-depth analysis of the molecular consequences of T1DM in tissue and of hyperglycemia in the cells. In addition, the potential influence of confounders, such as fetal sex and maternal metabolic factors other than glucose, could not be investigated in sub-analyses. Thus, further studies should not only confirm our findings in a larger first trimester cohort but also in trophoblasts isolated from placentas of pregnancies complicated with T1DM. Likewise, other metabolic derangements associated with T1DM such as dyslipidemia should also be considered. 

## 4. Materials and Methods

### 4.1. First Trimester Placental Tissue Collection 

The study was approved by the institutional review board and ethical committee of the Medical University of Graz (23e435 ex 10/11). Women participating in the study signed a written informed consent. First trimester placental tissue (gestational week 5–12) was obtained after elective pregnancy termination for psychosocial reasons, washed with phosphate-buffered saline (PBS), and immediately used for trophoblast isolation, cryopreserved at −80 °C until further use, or fixed in 4% PFA and paraffin-embedded.

### 4.2. First Trimester Trophoblast Isolation 

Primary human first trimester trophoblasts were isolated as previously described [33,41]. Briefly, first trimester placental tissue was digested with Dispase/DNAse and Trypsin (Gibco, Invitrogen, Carlsbad, CA, USA). After Percoll (Gibco) gradient centrifugation, fibroblasts and common leukocyte-antigen expressing cells were removed by negative immunoselection with anti-CD90 (Dako, Glostrup, Denmark) and anti-CD45 conjugated magnetic beads (Thermo Fisher Scientific, Rockford, IL, USA), respectively. Purity of the trophoblast preparations was determined by human chorionic gonadotropin secretion as well as positive cytokeratin 7 (Dako, 1:750) and negative vimentin (Dako, 1:250) immunostaining. Only isolations with a purity ≥95% were used.

### 4.3. Protein Array 

The level of 60 cell-cycle-related proteins was determined in first trimester placental tissue from control and T1DM pregnancies using the Cell Cycle Control Antibody Array (Fullmoon Biosystems, Sunnyvale, CA, USA). Clinical data are shown in Table 1. Gestational age was determined by ultrasound measurement of crown rump length. Type 1 diabetes mellitus (T1DM) was defined by glycosylated hemoglobin (HbA1c) levels ≥6.0% in the first trimester.

Briefly, 10–40 mg of placental tissue was lysed in extraction buffer (Fullmoon Biosystems, Sunnyvale, CA, USA) using a tissue lyser (MagNa Lyser, Roche, Basel, Switzerland). Protein concentration was determined using the bicinchoninic acid assay (Thermo Fisher Scientific, Rockford, IL, USA). The array slides containing antibodies against the proteins of interest were pre-treated with blocking solution (Fullmoon Biosystems, Sunnyvale, CA, USA) for 30 min at room temperature. Subsequently, the slides were incubated with 75 µg of biotin-labeled protein lysates at 4 °C overnight. Unbound proteins were removed with washing solution (Fullmoon Biosystems, Sunnyvale, CA, USA) and proteins were detected using Cy3-conjugated streptavidin (Sigma Aldrich, St. Louis, MO, USA). Signal intensity was calculated using GenePix Pro 6.0 software (Molecular Devices, San Jose, CA, USA). Data were normalized to the median intensity of all antibodies on the array after background subtraction.

### 4.4. Ki67 Immunolocalization 

Ki67 immunostaining was performed on 3 µm thick sections of paraffin-embedded first trimester placental tissue as previously described [6]. Briefly, after deparaffinization and antigen retrieval in 10 mM citrate buffer (pH 6) at 110 °C for 10 min, Ki67 immunohistochemistry was performed using the UltraVision LP Detection System (HRP polymer kit, Thermo Fisher Scientific, Rockford, IL, USA) as per manufacturer’s guidelines. Endogenous peroxidase was blocked with UltraVision Hydrogen Peroxide Block for 10 min at room temperature followed by a 5-min incubation with UltraVision Protein. Anti-Ki67 antibody (1:100, BD-Biosciences, Bedford, MA, USA) was diluted in Antibody Diluent (Dako, Glostrup, Denmark) and incubated for 1 h at room temperature in a humidified chamber. Antibody detection was conducted with Primary Antibody Enhancer using an HRP-labeled polymer (Dako, Glostrup, Denmark) and AEC Single Solution (Thermo Scientific, Rockford, IL, USA). Nuclei were counterstained with Mayer’s hematoxylin (Gatt Koller, Absam, Austria), and images were acquired with a Zeiss Axio Z1 microscope (Zeiss, Oberkochen, Germany) equipped with a digital camera (Olympus X, Tokyo, Japan) using the AxioVision Software (Zeiss, Oberkochen, Germany).

### 4.5. Trophoblast Cell Number Determination and FACS Analysis 

Trophoblasts were cultured in Dulbecco’s Modified Eagle medium (DMEM, Gibco) supplemented with 1% (*v*/*v*) penicillin/streptomycin (Gibco) in a humidified incubator at 37 °C and 5% CO2. Cells (1 × 10^6^ per well) were incubated in the absence (control) or presence of 25 mM D-glucose (Sigma Aldrich, St. Louis, MO, USA; hyperglycemia) and 5.5 mM D-glucose + 19.5 mM D-mannitol (Sigma Aldrich, St. Louis, MO, USA; osmotic control) for 48 h. Thereafter, cells were enzymatically detached using Trypsin-EDTA (Gibco) and the trophoblast cell number was counted using the CASY TCC Cell Counter and Analyzer System (Schärfe System, Reutlingen, Germany), which allows discrimination between viable and dead cells [42]. Trophoblasts were also stained with DAPI (Thermo Fisher Scientific, Rockford, IL, USA) for FACS analysis. The percentage of cells in each phase of the cell cycle was analyzed using MultiCycle AV Software (Phoenix Flow Systems, San Diego, CA, USA). 

### 4.6. Invasion Assay 

Trophoblast invasion was measured using 12 mm Transwell inserts with 12 μm pores (Millipore, Billerica, MA, USA). The inserts were pre-coated with rat-collagen I, mouse-collagen IV, mouse-laminin, and human-fibronectin (BD-Biosciences, Bedford, MA, USA), and placed into a 24-well plate containing DMEM supplemented with 10% (*v*/*v*) fetal calf serum (FCS, Thermo Fisher Scientific, Rockford, IL, USA). 

Primary first trimester trophoblasts (5 × 10^4^ per Transwell) were pre-labeled with 3[H]-5-methyl-thymidine (GE Healthcare, Chicago, IL, USA) overnight. Subsequently, cells were resuspended in DMEM without FCS and seeded in the upper chamber of the inserts in absence (control) or presence of 25 mM D-glucose (hyperglycemia) or 5.5 mM D-glucose + 19.5 mM D-mannitol (osmotic control) as described above. After 48 h, cells in the upper chamber were detached using Trypsin-EDTA. The membrane of the insert containing the invading cells was carefully removed with a scalpel and added to the medium in the lower chamber. In both fractions, radioactive disintegrations were counted in a scintillation counter (Beckman Coulter, Brea, CA, USA). The invasion index was calculated as counts per minute (cpm) in the lower chamber and the membrane divided by the total cpm (upper chamber + lower chamber + membrane). 

### 4.7. Statistical Analysis 

GraphPad Prism 8 and SPSS Statistics 25 was used for statistical analyses. Sample size precluded data analysis for normal distribution. Therefore, statistical significance of group differences was robustly determined by a Mann Whitney U Test, whereas treatment effects were determined in a paired analysis using a Wilcoxon signed-rank test. Associations between cell-cycle-related protein levels and gestational age were analyzed by Spearman’s correlation coefficient. *p* < 0.05 was considered statistically significant. 

## Figures and Tables

**Table 1:** Characteristics of the study cohort used in the protein array.

	Control (*n* = 11)	T1DM (*n* = 12)	*p* Value
**Gestational age** (weeks)	7.6 ± 1.0	8.3 ± 1.4	n.s.
**Maternal age** (years)	30.3 ± 6.7	30.5 ± 5.2	n.s.
**HbA1c** (%)	n.d.	7.7 ± 1.6	n.d.
**Maternal BMI** (kg/m^2^)	24.2 ± 2.5	22.9 ± 1.1	n.s.

Mann Whitney U-test. n.s. = not significant; n.d. = not determined; data were not available for all women.

## Data Availability

The data presented in this study are available on request from the corresponding author. The data are not publicly available due to privacy/ethical restrictions.
